# PeptX: Using Genetic Algorithms to optimize peptides for MHC binding

**DOI:** 10.1186/1471-2105-12-241

**Published:** 2011-06-17

**Authors:** Bernhard Knapp, Verena Giczi, Reiner Ribarics, Wolfgang Schreiner

**Affiliations:** 1 Center for Medical Statistics, Informatics and Intelligent Systems, Department for Biosimulation and Bioinformatics, Medical University of Vienna, Vienna, Austria; 2 University of Applied Sciences, FH Campus Wien, Department of Bioengineering, Vienna, Austria

## Abstract

**Background:**

The binding between the major histocompatibility complex and the presented peptide is an indispensable prerequisite for the adaptive immune response. There is a plethora of different *in silico *techniques for the prediction of the peptide binding affinity to major histocompatibility complexes. Most studies screen a set of peptides for promising candidates to predict possible T cell epitopes. In this study we ask the question vice versa: Which peptides do have highest binding affinities to a given major histocompatibility complex according to certain *in silico *scoring functions?

**Results:**

Since a full screening of all possible peptides is not feasible in reasonable runtime, we introduce a heuristic approach. We developed a framework for Genetic Algorithms to optimize peptides for the binding to major histocompatibility complexes. In an extensive benchmark we tested various operator combinations. We found that (1) selection operators have a strong influence on the convergence of the population while recombination operators have minor influence and (2) that five different binding prediction methods lead to five different sets of "optimal" peptides for the same major histocompatibility complex. The consensus peptides were experimentally verified as high affinity binders.

**Conclusion:**

We provide a generalized framework to calculate sets of high affinity binders based on different previously published scoring functions in reasonable runtime. Furthermore we give insight into the different behaviours of operators and scoring functions of the Genetic Algorithm.

## Background

Antigen presenting cells (APCs) present peptides via their major histocompatibility complex (MHC) to T cell receptors (TCRs) of T cells which play an essential role in the adaptive immune system [1]. Before any recognition between T cell and APC can take place the peptides need to be processed within the APC and afterwards presented in a stable way on the cell surface of the APC. For most of these steps ample prediction methods exist [2-5]. In this context the binding prediction between MHC and the presented peptides is usually referred as T cell epitope prediction. In a usual workflow one wants to test different peptides or even possible fragments of a whole protein for its binding affinity to a given MHC. After this rough pre-selection step the most promising candidates are then tested in wet-lab experiments for their definitive binding affinity and applicability. The success rate of these approaches is discussed abundantly in the literature: While it is known that there is still much space for improvement of B cell epitope prediction methods [6] and that MHC class I T cell epitope prediction works quite well [2,7-9], the opinions on the success of MHC class II T cell epitope prediction differ. Various reviews conclude that it lacks reliability [10-13], though others are more enthusiastic about the results obtainable with MHC class II prediction tools [3].

While most methods are sequence-based and it is generally believed that sequence-based approaches are more successful [8], there were also some structure-based approaches reported [14]. They range from molecular dynamics (MD) methods [15-17] over quantitative structure-activity relationship (QSAR) [18] methods to empirical scoring methods [19-21].

By contrast, when asking the question vice versa the scientific challenge changes: One does not want to test a specific set of peptides for their binding affinities, but knows a specific MHC allele and wants to determine a set of peptides which have a very high binding affinity to this MHC allele. Applications could be found for example in peptide immunotherapy (PIT), in allergy [22-24], cancer therapy [25], or peptide vaccines [26] by indentifying altered peptide ligands with favourable properties for the respective purpose.

Given the fact that for MHC class I 20^9 ^and for MHC class II 20^18 ^or even more different peptides exist, it is obvious that not each individual peptide can be tested. This limitation also includes *in silico *techniques since even if we assume that modern computers can test 10 peptides per second it would still take more than 1 600 years (20^9^/(10×60×60×24×365)) to predict the binding affinity for all theoretically possible peptides binding to one single MHC class I allele. The runtime would further increase if the effect on the interaction between peptide/MHC (pMHC) and TCR would additionally be predicted.

Several approaches to address the challenge of optimization for pMHC/TCR interaction were reported: In early methods Alexander et al. employed the main HLA-DR anchors to increase the peptide/MHC binding affinity as well as the use of bulky and charged residues to increase T cell recognition [27,28]. Shang et al. used computational alanine scanning to indentify hotspots which were then systematically substituted and scored to optimize a tumor immunodominant epitope [29]. Also for the peptide and MHC interaction several approaches were reported: Reche et al. published a webserver for the formulation of multi-epitope vaccines [30]. Toussaint et al. developed a mathematical framework on the basis of integer linear programming to obtain good candidates for epitope-based vaccines. In this context they define good as a combination of mutation tolerance, allele coverage, antigen coverage, and antigen processing [31]. Contrary, Parker et al. implemented an optimization algorithm to find point mutations to reduce the immunogenicity of a protein while maintaining stability and function [32]. Lazar et al. presented an optimization approach for antibody humanization [33]. Bhasin et al. reported quantitative matrices on the basis of binding and non binding peptides sets [34]. Similarly, in the studies of Doytchinova et al. and Walshe et al. the preferred amino acids for peptide positions were selected based on experimental data sets. This leads to a set of a few hundred peptides which were then evaluated in silico and experimentally [35,36]. Guan et al. reported amino acid descriptors to characterize the interaction between peptide and MHC. On this basis of the defined binding model high-affinity binders were designed [37].

However, in these approaches the search space for optimal binders is frequently reduced by an initial selection of preferred amino acids for several positions. Given this limitation and the fact that the total search space would be 20^9 ^combinations we propose the use of a Genetic Algorithm (GA) to be able to investigate the whole search space in justifiable runtime. Hereby the task reformulates to finding the most efficient and reliable modes of GAs. Hence, we implemented a framework "PeptX" for optimizing GAs for the prediction of sets of peptides with high binding affinities within reasonable runtime. By means of the framework "PeptX" we evaluated which combination of parameters and operators yields the most rapid convergence of the GA towards a set of high binding affinity peptides.

It is not the major aim of this study to compare different scoring functions since this issue was already addressed several times in the recent literature e.g. MHC class I [9,38-41] and MHC class II [3,10-13]. Even a "machine learning in immunology competition" was organized for this purpose (http://www.kios.org.cy/ICANN09/MLI.html). Since there is already a plethora of benchmarks for prediction methods our study is focused on a generalized approach on how to predict huge sets of high affinity binders on the basis of these previously published methods.

## Methods

**Figure 1:**
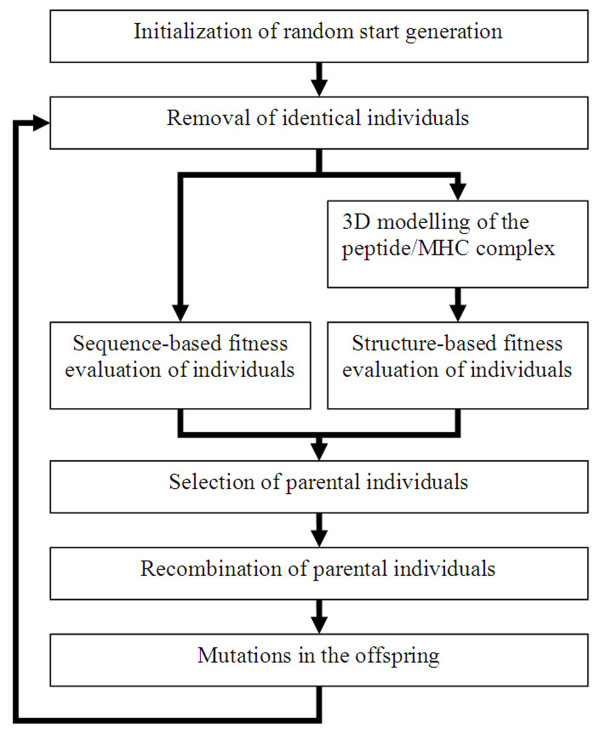
**Workflow of the GA**.

We implemented the C++ framework "PeptX" for GAs to optimize a set of peptides for MHC binding and tested it for HLA-A*02:01. We implemented the whole framework in an object-oriented way which makes it easily maintainable and extendable. The workflow of the GA is illustrated in Figure 1 and described in detail in the subsequent sections.

### Initialization of random start generation and removal of identical individuals

We generated a random set of peptides as initial generation. Each individual consisted of a string of 9 characters, each of them representing one position in the peptide. Therefore, we randomly selected for each position in the peptide one of the 20 essential amino acids. To avoid too dominant individuals (genetic drift) we purged the whole population from identical peptides at each generation. The removed individuals were replaced by new random ones. This purging step ensures that the algorithm will not get stuck in a local optimum and the genetic diversity will remain over the whole runtime of the GA.

### Fitness evaluation of the individuals

In the next step we assigned a fitness value to each of the peptides by employing a scoring function for peptide/MHC binding. We utilized the following sequence-based scoring functions: Immune Epitope Database (IEDB) [42], SYFPEITHI [43], SVMHC [44], SVRMHC [45]. Additionally, we employed the structure-based scoring function XScore [46] in combination with the threading technique of SCATD [47] for the construction of the 3D peptide/MHC model. This combination was found most appropriate for structural peptide/MHC binding predictions [40,48].

### Selection of parent individuals

Individuals were selected as parents for the next generation based on the previously described fitness evaluation. We implemented 7 different selection methods according to the literature which are briefly summarized in the following. For details of the single algorithms we refer to [49-51]:

1. Proportional (roulette wheel) selection: To each individual an area on a roulette wheel is assigned depending on its fitness value. Individuals with higher value have a higher probability to be selected than individuals with lower fitness value.

2. Linear rank selection: The individuals obtain a rank in correspondence to their fitness value. The selection is performed on the basis of this rank.

3. Binary tournament selection: Two randomly selected individuals compete for their selection where the one with the higher fitness value wins without any other stochastic influences.

4. Random selection: The individuals are selected in a completely random way.

5. Best percent selection: The n best percent of the population are chosen straight forwardly.

6. Q-tournament selection: All individuals participate in q tournaments, where the individuals with the most victories are selected.

7. Stochastic universal sampling: Similar to the proportional selection, every individual obtains a segment on a roulette wheel according to its fitness value. However, it is turned only one time with n-balls where n is the number of individuals in the population.

### Recombination (crossover) of parent individuals

To recombine the previously selected parent individuals we implemented 6 different crossover operators according to [51,52]:

1. Single point crossover: The amino acid sequence of parent 1 (P1) and parent 2 (P2) are cleaved at one randomly chosen position. The four parts of P1 and P2 are recombined at the cleavage point in a way that each child contains one part of P1 and the other part of P2.

2. Double point crossover: P1 and P2 are recombined at two randomly chosen positions.

3. Distance bisector crossover: P1 and P2 are recombined in the middle (e.g. for MHC class I: position 5 of the peptide).

4. Multi point crossover: P1 and P2 are recombined at r randomly chosen positions where r itself is also a random number (r ≤ 9).

5. Uniform crossover: Related to the multi-point crossover, however, each position within the sequence obtains a randomly assigned probability for recombination. If this probability exceeds a certain threshold, a recombination at this position occurs.

6. Shuffle crossover: Similar to the double point crossover P1 and P2 are recombined at two randomly selected positions. However, before recombination the amino acids are shuffled in both parents. After recombination the amino acids are unshuffled.

### Mutations in the offspring

To maintain genetic diversity we performed mutations on each offspring generation. For this purpose we implemented 2 different operators [51]:

1. "Single point amino acid mutation": One amino acid is substituted randomly by another amino acid which belongs to another amino acid class (unpolar, polar, basic, or acid).

2. "Single point nucleobase mutation": A mutation is introduced in the triplet of the genetic code. Stop codons are avoided.

We applied the mutation with a probability of 3% for each amino acid position.

### Stop criterion

Although it turned out that the most reasonable combinations of parameters let the GA converge much earlier (see result section) we always simulated 50 generations, so as to allow for comparability between the different parameterizations.

## Results and Discussion

### Operators and their combinations

**Figure 2:**
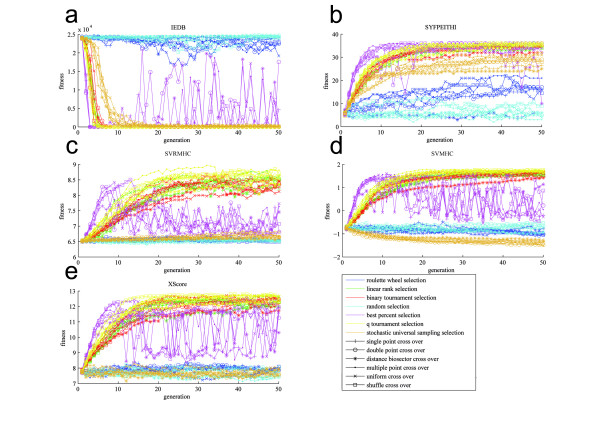
**Median fitness score of the individuals over generations**. For each generation we calculate the median of the fitness scores of the 100 individuals per generation. At generation 1 the individuals are random while at generation 50 the individuals should be optimized according to the corresponding scoring function: (a) IEDB, (b) SYFPEITHI, (c) SVRMHC, (d) SVMHC and, (e) XSCORE. For all scoring functions except IEDB the optimum is a maximum (higher values indicate a higher binding affinity, e.g. pIC50-value). In IEDB the optimum is a minimum (lower values indicate a higher binding affinity, e.g. IC50-value). The selection operators are grouped by colour while the recombination operators are grouped by marker symbols. Only the data for the "single point amino acid mutation" is shown.

In total we evaluated 7 selection operators, 6 recombination operators, 5 fitness functions, and 2 mutation operators in all possible combinations. This yields 420 (7 × 6 × 5 × 2) independent runs of the GA. In each run we calculated 50 generations, each generation consisting of 100 individuals (peptides). Therefore, in total we performed 2 100 000 (420 × 50 × 100) different peptide evaluations. The results are illustrated in Figure 2 and will be summarized in the subsequent sections grouped by selection operator, recombination operator and scoring function. Since Figure 2 contains a plethora of data we additionally depict several numerical details of these data in additional file 1.

### Selection operators have a strong influence on the convergence

It can be seen that most operator combinations work well and converge within the first 10 to 20 generations. However, there are a few exceptions: Obviously, a random selection does not improve fitness in an evolutionary process. We included this operator only as negative control. Additionally, the roulette wheel selection operator is, in its basic implementation, not able to generate enough evolutionary pressure to lead the population to convergence. This can be explained by the fact that, in the present setting, the whole roulette wheel was divided into small sections for each individual where the area of the section is proportional to the fitness value. The theoretical maximum for IC50 values is at around 50 000 while binding peptides usually have IC50 values below 500. Therefore, the area for a binding individual is not much larger than for non binders closely above 500. These proportions of the roulette wheel seem to hamper the evolutionally pressure on the population. A possibility to improve the performance of the roulette wheel selection operator would be a transformation of the distribution of the IC50 values. In contrast, the best percent selection usually converges fastest, however, it seems that the genetic pressure is too strong and the population loses the genetic diversity after reaching a certain optimum and cannot deal with the random mutations and removal of identical individuals anymore. Therefore, after a very fast convergence of the best percent selection between generation 1 and 10 the results are becoming worse between generations 10 and 20. Quite good results are usually achieved by the tournament selection operators. Although they are converging slightly slower than the best percent selection, they reach a better and more stable optimum.

### Recombination operators have minor influence on the convergence

The influence of recombination operators on the convergence of the population is limited. The results strongly depend on the interaction with the selection operator. There are examples for a good convergence as well for a bad one for nearly each of the recombination operators. The only exception is the distance bisector cross over whose convergence is never among the top performers. This result was to be expected since if the peptide is always cleaved exactly in the middle it takes much longer to find the global optimum.

### Different mutation operators did not have a significant influence on the population

The usage of "single point amino acid mutation" versus "single point nucleobase mutation" method did not influence the convergence of the populations. Operators converging with the "single point amino acid mutation" did also converge with the "single point nucleobase mutation". The same also applies for non converging operators. Hence, we show only the data for the "single point amino acid mutation" in Figure 2.

### Different scoring functions lead to diverse sets of optimized peptides

**Figure 3:**
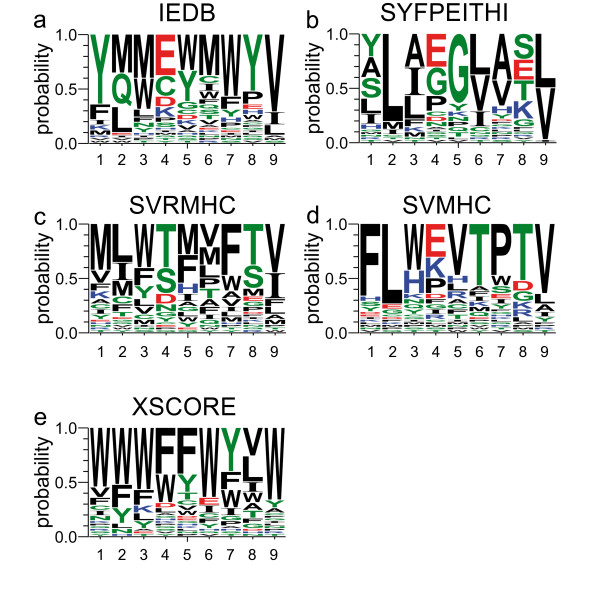
**Sequence logos of the best individuals**. Logos created by WebLogo [56] on the basis of the best individuals of generation number 50 of each operator combination grouped by scoring function: (a) IEDB, (b) SYFPEITHI, (c) SVRMHC, (d) SVMHC and, (e) XSCORE.

The most severe influence is created by the scoring function used for the calculation of the fitness values. Although it is not purposeful to compare the convergence between different scoring functions, it is interesting to investigate individuals which are predicted as optimal for HLA-A*02:01 by different scoring functions (see Figure 3). In the subsequent paragraphs they are discussed and compared to experimentally known anchor residues determined by Falk et al. [53]. These residues are described in the SYFPEITHI [43] database as L or M for position 2, L or V for position 9, and V as auxiliary anchor for position 6.

#### IEDB

At positions 1, 7, 8 and 9 the scoring function exhibits a clear preference for one specific amino acid. The preferred residues for the anchor positions 2 and 9 are in agreement with the experimental data. In contrast, the algorithm also proposed Q, a rather polar amino acid, for position 2. Furthermore, it is noteworthy that there is a tendency towards amino acids with a higher Van-der-Waals volume. The consensus sequence would exhibit a volume of 1 194 Å^3^.

#### SYFPEITHI

The SYFPEITHI scoring function tends to prefer small, polar, and apolar amino acids while retaining a rather low background noise. The anchors at positions 2, 6 and 9 are clearly the same as described in the literature, however, this is not surprising since the SYFPEITHI database is partly based on the data of Falk et al. [53]. The Van-der-Waals volume of the consensus sequence is 877 Å^3^.

#### SVRMHC

This scoring function exhibits a rather promiscuous impression. There is no strong preference for a single residue. However, also here L and M are strong at position 2 while L and V are present in position 9. Besides, most of the remaining top performing residues for position 9 fall into the same class as V or L. The Van-der-Waals volume of the consensus sequence is 1 066 Å^3^.

#### SVMHC

While most of the time the SVMHC scoring function prefers one single very dominant amino acid per position, the algorithm shows almost never a clear "second place". One residue dominates and the remaining ones are more or less random. Interesting is that at position 3, the algorithm mixes an apolar (W) residue with a charged one (H). Despite this fact, there arises a pattern, because both amino acids carry aromatic residues that seem to be important for position 3 according to SVMHC. Also the other algorithms, with the exception of SYFPEITHI, support this preference. The consensus sequence of SVMHC matches the experimental anchor residues. However, V at position 6 is not present. The Van-der-Waals volume of the consensus sequence is 1 017 Å^3^.

#### XSCORE

XSCORE shows a strong tendency to assert high scores to big, hydrophobic amino acids. The dominating residue at position 1, 2, 3, 5, and 9 is W, whereas at position 5 and 7 F and Y are preferred. The corresponding consensus sequence would be repetitive and the probability of occurrence in the genetic code would be very low. The consensus sequence yields a Van-der-Waals volume of 1 331 Å^3^. The theoretical maximum (poly W-peptide) for a nonamer would only be slightly above at 1 467 Å^3^. One explanation for this exceptional behaviour could be the fact that XSCORE is the only structural scoring function used. Results suggest that XSCORE tries to fill the MHC binding groove as tightly as possible.

### Comparison of the optimized peptides with public experimental data

One question which arises when having a look at the optimized peptides is: Are the predicted peptides related to training sets used by the respective scoring functions? While this is an interesting question, it is hard to give a definitive answer since even if the training data set is given in the publication of the scoring function, it is likely that the scoring function was re-calibrated with new data in the meantime since publication. For this reason we compared the resulting top 210 peptides (7×6×5) with the 14828 experimental peptide binding affinities available from the IEDB [54]. Interestingly, no peptide was identical. Only 2 peptides had a Levenshtein-distance of 2. Those 2 peptides are experimentally determined high affinity binders. All other peptides are more different from the public data. This would suggest that the scoring functions predict novel peptides by the use of the GA. However, as already mentioned above we cannot guarantee that the scoring functions were not trained on further "private" datasets.

### Validation of the 5 consensus peptides via experimental binding assays

Since a validation on the basis of existing public experimental data was not possible we were able to find a co-operation partner who tested our consensus peptides for experimental peptide/MHC binding affinity. This was done at the revision level of the manuscript hence it is evident that the whole prediction was validated.

Although at first sight it seemed that the consensus peptides will be difficult to synthesise because of the multiple W and M residues, all peptides could be synthesised experimentally. All 5 consensus peptides are high affinity binders. The detailed results are shown in Table 1.

**Table 1:** Experimental validation of the consensus peptides.

YMMEWMWYV	< 0.5 nM
YLAEGLASL	1.3 nM

MLWTMVFTV	< 0.5 nM

FLWEVTPTV	1.1 nM

WWWFFWYVW	45 nM

The affinities are expressed as IC50 values. Values below 0.5 nM are beyond the range of sensitivity of the assay and therefore reported as < 0.5 nM.

## Conclusion

We developed the framework "PeptX" for the evaluation of different operators for GAs in the context of peptide/MHC binding optimization. At the current time the focus of this study is mainly on the information theoretical part and may not be directly applicable in the wet-lab e.g. for questions like "what is the set of peptides (from a relevant pathogen), that bind an MHC with high affinity". However, in a next step further applications can be found in peptide immunotherapy: One wants to find high affinity binding peptides, but with certain constraints in the sequence to avoid allergic reactions (see Introduction).To be further able to address this issue we evaluated different parameter-sets of Genetic Algorithms in relation to MHC. On this basis further studies with direct relation to the wet-lab can be carried out.

The work most similar to our study was published by Wisniewska and co-workers. They combined an ant colony optimization algorithm with an artificial neural network classifier to iteratively adapt octapeptides for MHC class I stabilization [55]. However, to our knowledge our study is the first study which investigates the operators of a GA in relation to maximizing peptide/MHC binding affinity.

On the basis of our study we found two remarkable characteristics in the evolutionary process of the individuals (peptides). Firstly, it is intriguing that although selection operators have a strong influence on the convergence of the population while recombination operators have only minor influence, most reasonable operator combinations lead to convergence of the population. Long before generation 50 an optimum for most of the populations is found (see Figure 2). The fastest convergence is usually achieved by the best percent selection; however, the tournament selections often provide a slightly better optimized median score. Secondly, the most crucial step is the choice of a scoring function appropriate for the particular investigation. All other operators have minor influence on what is finally found optimal for binding to a specific MHC. Although there are similarities between the consensus sequences of the scoring functions (see Figure 3), one obtains different sets of optimal peptides by each scoring function. Hence, choosing a scoring function which is suboptimal for the purpose of the respective study renders almost all other parameters of the GA irrelevant. One should choose the employed scoring function with caution on the basis of previously published benchmarks (see Introduction). However, the GA itself can be utilized with an arbitrary scoring function and the convergence will mainly depend on the parameters evaluated in this study.

The PeptX framework is available for download including C++ source code for Linux at http://www.meduniwien.ac.at/msi/md/sourceCodes/peptX/peptX.htm The download and usage is for free for academic researchers.

## Competing interests

The authors declare that they have no competing interests.

## Authors' contributions

BK designed the study, carried out programming work and drafted the manuscript. VG carried out programming work and helped to draft the manuscript. RR helped in the analysis of data and revised the manuscript critically. WS supervised the research and revised the manuscript critically. All authors read and approved the final manuscript.

## Supplementary Material

Additional file 1 **Numerical details of individual operator combinations**. This file contains the numerical details of Figure 2. The median value over the first 10 generations, the median value over the last 10 generations, the maximum median, and the minimum median is shown. Click here for file
