# Supplementary material for: PeptX: Using Genetic Algorithms to optimize peptides for MHC binding
Source: BMC Bioinformatics. 2011 Jun 17;12:241. doi: 10.1186/1471-2105-12-241 (PMC3225262; doi:10.1186/1471-2105-12-241)
Supplement: Additional file 1 — Numerical details of individual operator combinations. This file contains the numerical details of Figure 2. The median value over the first 10 generations, the median value over the last 10 generations, the maximum median, and the minimum median is shown. [file 1471-2105-12-241-S1.DOC]

Numerical details of individual operator combinations.

| **Scoring function** | **Selection operator** | **Crossover operator** | **Median last 10 generations** | **Median first 10 generations** | **Highest median** | **Lowest median** |  | **Scoring function** | **Selection operator** | **Crossover operator** | **Median last 10 generations** | **Median first 10 generations** | **Highest median** | **Lowest median** |
| --- | --- | --- | --- | --- | --- | --- | --- | --- | --- | --- | --- | --- | --- | --- |
| IEDB | SRW | CSP | 23667.20 | 23664.70 | 24327.40 | 21979.40 |  | SYFPEITHI | SRW | CSP | 15.00 | 8.00 | 16.00 | 6.00 |
| IEDB | SRW | CDP | 21900.10 | 24310.67 | 24458.00 | 20274.70 |  | SYFPEITHI | SRW | CDP | 17.00 | 7.00 | 18.00 | 6.00 |
| IEDB | SRW | CDB | 24081.75 | 23937.25 | 24400.60 | 19634.45 |  | SYFPEITHI | SRW | CDB | 5.00 | 5.00 | 6.00 | 3.00 |
| IEDB | SRW | CMP | 21614.65 | 24264.13 | 24510.05 | 19713.45 |  | SYFPEITHI | SRW | CMP | 21.00 | 12.00 | 22.00 | 6.00 |
| IEDB | SRW | CUF | 21442.15 | 24247.25 | 24429.45 | 16577.60 |  | SYFPEITHI | SRW | CUF | 16.00 | 11.50 | 21.50 | 6.00 |
| IEDB | SRW | CSF | 22977.80 | 23896.50 | 24350.55 | 18812.50 |  | SYFPEITHI | SRW | CSF | 17.00 | 8.00 | 22.00 | 6.00 |
| IEDB | SLR | CSP | 5.51 | 1740.39 | 23991.60 | 5.47 |  | SYFPEITHI | SLR | CSP | 34.00 | 23.00 | 35.00 | 6.00 |
| IEDB | SLR | CDP | 5.50 | 119.12 | 24089.55 | 5.46 |  | SYFPEITHI | SLR | CDP | 36.00 | 21.00 | 36.00 | 6.00 |
| IEDB | SLR | CDB | 5.75 | 50.84 | 23996.15 | 5.70 |  | SYFPEITHI | SLR | CDB | 33.00 | 17.50 | 34.00 | 6.50 |
| IEDB | SLR | CMP | 5.57 | 771.60 | 24254.70 | 5.53 |  | SYFPEITHI | SLR | CMP | 36.00 | 19.50 | 36.00 | 6.50 |
| IEDB | SLR | CUF | 5.53 | 189.56 | 24001.80 | 5.48 |  | SYFPEITHI | SLR | CUF | 34.00 | 19.50 | 35.00 | 7.00 |
| IEDB | SLR | CSF | 5.52 | 32.75 | 24041.00 | 5.50 |  | SYFPEITHI | SLR | CSF | 34.00 | 17.50 | 35.00 | 7.00 |
| IEDB | SBT | CSP | 5.52 | 1273.28 | 23918.00 | 5.51 |  | SYFPEITHI | SBT | CSP | 36.00 | 21.50 | 36.00 | 6.00 |
| IEDB | SBT | CDP | 5.58 | 3399.03 | 24006.95 | 5.55 |  | SYFPEITHI | SBT | CDP | 34.00 | 19.50 | 35.00 | 5.50 |
| IEDB | SBT | CDB | 5.77 | 1578.58 | 24027.80 | 5.70 |  | SYFPEITHI | SBT | CDB | 32.00 | 20.50 | 32.00 | 7.00 |
| IEDB | SBT | CMP | 5.64 | 44.31 | 24085.75 | 5.61 |  | SYFPEITHI | SBT | CMP | 35.00 | 21.25 | 35.00 | 7.00 |
| IEDB | SBT | CUF | 5.48 | 674.48 | 24184.55 | 5.47 |  | SYFPEITHI | SBT | CUF | 34.00 | 19.75 | 35.00 | 6.00 |
| IEDB | SBT | CSF | 5.49 | 22.56 | 23804.70 | 5.47 |  | SYFPEITHI | SBT | CSF | 35.00 | 18.25 | 35.00 | 6.00 |
| IEDB | SRD | CSP | 24318.90 | 24085.28 | 24502.10 | 23728.45 |  | SYFPEITHI | SRD | CSP | 8.00 | 5.00 | 9.00 | 4.00 |
| IEDB | SRD | CDP | 24600.65 | 24353.90 | 24713.35 | 21571.35 |  | SYFPEITHI | SRD | CDP | 6.00 | 4.25 | 8.00 | 3.50 |
| IEDB | SRD | CDB | 24517.00 | 24144.38 | 24623.10 | 23910.95 |  | SYFPEITHI | SRD | CDB | 5.50 | 5.75 | 7.00 | 4.00 |
| IEDB | SRD | CMP | 24710.50 | 24309.75 | 24765.50 | 23961.75 |  | SYFPEITHI | SRD | CMP | 3.00 | 6.00 | 7.00 | 2.00 |
| IEDB | SRD | CUF | 24142.60 | 24236.08 | 24389.70 | 23097.15 |  | SYFPEITHI | SRD | CUF | 4.00 | 4.50 | 6.00 | 3.00 |
| IEDB | SRD | CSF | 23916.85 | 23893.88 | 24420.75 | 20181.75 |  | SYFPEITHI | SRD | CSF | 9.00 | 9.00 | 12.50 | 6.00 |
| IEDB | SBP | CSP | 6.77 | 6.30 | 24224.35 | 5.58 |  | SYFPEITHI | SBP | CSP | 33.00 | 26.50 | 34.00 | 5.00 |
| IEDB | SBP | CDP | 18.18 | 6.07 | 23836.40 | 5.51 |  | SYFPEITHI | SBP | CDP | 34.00 | 26.50 | 36.00 | 5.00 |
| IEDB | SBP | CDB | 139.38 | 43.46 | 23839.15 | 6.11 |  | SYFPEITHI | SBP | CDB | 26.00 | 27.00 | 35.00 | 7.50 |
| IEDB | SBP | CMP | 4356.76 | 6.45 | 24077.75 | 5.63 |  | SYFPEITHI | SBP | CMP | 36.00 | 30.00 | 36.00 | 5.00 |
| IEDB | SBP | CUF | 40.28 | 6.71 | 23939.15 | 5.58 |  | SYFPEITHI | SBP | CUF | 34.00 | 31.00 | 36.00 | 5.00 |
| IEDB | SBP | CSF | 15361.10 | 6.82 | 24117.75 | 5.60 |  | SYFPEITHI | SBP | CSF | 36.00 | 28.00 | 36.00 | 5.00 |
| IEDB | SQT | CSP | 5.48 | 45.81 | 24013.75 | 5.46 |  | SYFPEITHI | SQT | CSP | 35.00 | 22.50 | 36.00 | 6.00 |
| IEDB | SQT | CDP | 5.47 | 538.49 | 24087.00 | 5.45 |  | SYFPEITHI | SQT | CDP | 36.00 | 23.00 | 36.00 | 6.00 |
| IEDB | SQT | CDB | 5.65 | 38.37 | 23796.90 | 5.60 |  | SYFPEITHI | SQT | CDB | 35.00 | 22.00 | 35.00 | 6.00 |
| IEDB | SQT | CMP | 5.46 | 57.48 | 23624.55 | 5.45 |  | SYFPEITHI | SQT | CMP | 36.00 | 23.50 | 36.00 | 6.50 |
| IEDB | SQT | CUF | 5.44 | 182.56 | 24250.25 | 5.43 |  | SYFPEITHI | SQT | CUF | 36.00 | 22.50 | 36.00 | 6.00 |
| IEDB | SQT | CSF | 5.45 | 57.65 | 23963.60 | 5.44 |  | SYFPEITHI | SQT | CSF | 35.00 | 21.50 | 35.00 | 4.50 |
| IEDB | SSU | CSP | 113.22 | 10531.66 | 23884.15 | 47.39 |  | SYFPEITHI | SSU | CSP | 26.00 | 20.00 | 27.00 | 6.00 |
| IEDB | SSU | CDP | 102.84 | 13959.48 | 23915.15 | 24.12 |  | SYFPEITHI | SSU | CDP | 29.00 | 16.50 | 29.00 | 6.00 |
| IEDB | SSU | CDB | 242.52 | 12383.70 | 24094.40 | 190.11 |  | SYFPEITHI | SSU | CDB | 24.00 | 19.00 | 25.00 | 6.00 |
| IEDB | SSU | CMP | 81.33 | 16426.78 | 23931.75 | 57.36 |  | SYFPEITHI | SSU | CMP | 31.00 | 19.00 | 32.00 | 6.00 |
| IEDB | SSU | CUF | 40.07 | 16937.93 | 23851.30 | 25.62 |  | SYFPEITHI | SSU | CUF | 30.00 | 14.50 | 31.00 | 6.00 |
| IEDB | SSU | CSF | 277.11 | 14695.65 | 23956.70 | 65.37 |  | SYFPEITHI | SSU | CSF | 29.00 | 15.50 | 29.00 | 6.00 |
|  |  |  |  |  |  |  |  |  |  |  |  |  |  |  |
| SVRMHC | SRW | CSP | 6.52 | 6.51 | 6.57 | 6.48 |  | SVMHC | SRW | CSP | -1.00 | -0.68 | -0.47 | -1.04 |
| SVRMHC | SRW | CDP | 6.55 | 6.51 | 6.60 | 6.49 |  | SVMHC | SRW | CDP | -0.97 | -0.76 | -0.69 | -1.07 |
| SVRMHC | SRW | CDB | 6.55 | 6.53 | 6.68 | 6.49 |  | SVMHC | SRW | CDB | -0.79 | -0.79 | -0.68 | -0.95 |
| SVRMHC | SRW | CMP | 6.53 | 6.53 | 6.56 | 6.49 |  | SVMHC | SRW | CMP | -0.90 | -0.85 | -0.75 | -0.93 |
| SVRMHC | SRW | CUF | 6.52 | 6.54 | 6.55 | 6.49 |  | SVMHC | SRW | CUF | -0.98 | -0.93 | -0.72 | -1.06 |
| SVRMHC | SRW | CSF | 6.49 | 6.56 | 6.64 | 6.49 |  | SVMHC | SRW | CSF | -0.97 | -0.72 | -0.69 | -1.04 |
| SVRMHC | SLR | CSP | 8.25 | 6.76 | 8.56 | 6.53 |  | SVMHC | SLR | CSP | 1.62 | 0.03 | 1.67 | -0.73 |
| SVRMHC | SLR | CDP | 8.51 | 6.81 | 8.59 | 6.52 |  | SVMHC | SLR | CDP | 1.56 | 0.21 | 1.64 | -0.77 |
| SVRMHC | SLR | CDB | 8.17 | 6.67 | 8.38 | 6.52 |  | SVMHC | SLR | CDB | 1.53 | 0.07 | 1.57 | -0.75 |
| SVRMHC | SLR | CMP | 8.71 | 6.84 | 8.80 | 6.51 |  | SVMHC | SLR | CMP | 1.68 | 0.06 | 1.75 | -0.73 |
| SVRMHC | SLR | CUF | 8.48 | 7.03 | 8.62 | 6.53 |  | SVMHC | SLR | CUF | 1.64 | 0.08 | 1.67 | -0.70 |
| SVRMHC | SLR | CSF | 8.50 | 6.73 | 8.53 | 6.51 |  | SVMHC | SLR | CSF | 1.60 | 0.25 | 1.63 | -0.71 |
| SVRMHC | SBT | CSP | 8.26 | 6.87 | 8.68 | 6.50 |  | SVMHC | SBT | CSP | 1.62 | 0.06 | 1.67 | -0.78 |
| SVRMHC | SBT | CDP | 8.20 | 6.81 | 8.41 | 6.53 |  | SVMHC | SBT | CDP | 1.47 | 0.06 | 1.54 | -0.75 |
| SVRMHC | SBT | CDB | 8.23 | 6.85 | 8.47 | 6.53 |  | SVMHC | SBT | CDB | 1.35 | 0.10 | 1.42 | -0.78 |
| SVRMHC | SBT | CMP | 8.47 | 6.81 | 8.54 | 6.49 |  | SVMHC | SBT | CMP | 1.62 | 0.17 | 1.67 | -0.76 |
| SVRMHC | SBT | CUF | 8.06 | 6.92 | 8.16 | 6.48 |  | SVMHC | SBT | CUF | 1.65 | 0.02 | 1.70 | -0.77 |
| SVRMHC | SBT | CSF | 8.46 | 6.82 | 8.66 | 6.50 |  | SVMHC | SBT | CSF | 1.60 | 0.04 | 1.66 | -0.73 |
| SVRMHC | SRD | CSP | 6.51 | 6.50 | 6.53 | 6.42 |  | SVMHC | SRD | CSP | -0.76 | -0.82 | -0.68 | -0.95 |
| SVRMHC | SRD | CDP | 6.56 | 6.51 | 6.60 | 6.47 |  | SVMHC | SRD | CDP | -0.66 | -0.79 | -0.55 | -0.88 |
| SVRMHC | SRD | CDB | 6.54 | 6.50 | 6.60 | 6.48 |  | SVMHC | SRD | CDB | -0.97 | -0.80 | -0.72 | -1.01 |
| SVRMHC | SRD | CMP | 6.54 | 6.53 | 6.58 | 6.51 |  | SVMHC | SRD | CMP | -0.95 | -0.72 | -0.68 | -1.01 |
| SVRMHC | SRD | CUF | 6.53 | 6.54 | 6.60 | 6.50 |  | SVMHC | SRD | CUF | -0.73 | -0.74 | -0.64 | -0.81 |
| SVRMHC | SRD | CSF | 6.53 | 6.50 | 6.59 | 6.44 |  | SVMHC | SRD | CSF | -0.57 | -0.65 | -0.51 | -0.70 |
| SVRMHC | SBP | CSP | 7.05 | 7.53 | 8.32 | 6.50 |  | SVMHC | SBP | CSP | -0.08 | 1.16 | 1.54 | -0.75 |
| SVRMHC | SBP | CDP | 6.81 | 7.48 | 8.27 | 6.51 |  | SVMHC | SBP | CDP | 0.85 | 0.73 | 1.58 | -0.75 |
| SVRMHC | SBP | CDB | 7.12 | 7.01 | 7.85 | 6.50 |  | SVMHC | SBP | CDB | 0.80 | 0.65 | 1.55 | -0.82 |
| SVRMHC | SBP | CMP | 7.06 | 7.56 | 8.15 | 6.51 |  | SVMHC | SBP | CMP | -0.05 | 1.29 | 1.56 | -0.65 |
| SVRMHC | SBP | CUF | 7.02 | 7.78 | 8.42 | 6.49 |  | SVMHC | SBP | CUF | -0.17 | 0.98 | 1.61 | -0.77 |
| SVRMHC | SBP | CSF | 6.81 | 7.76 | 8.51 | 6.54 |  | SVMHC | SBP | CSF | -0.16 | 1.20 | 1.59 | -0.79 |
| SVRMHC | SQT | CSP | 8.63 | 7.10 | 8.71 | 6.56 |  | SVMHC | SQT | CSP | 1.68 | 0.50 | 1.73 | -0.70 |
| SVRMHC | SQT | CDP | 8.52 | 6.91 | 8.71 | 6.50 |  | SVMHC | SQT | CDP | 1.69 | 0.19 | 1.74 | -0.80 |
| SVRMHC | SQT | CDB | 8.31 | 7.04 | 8.39 | 6.52 |  | SVMHC | SQT | CDB | 1.65 | 0.39 | 1.70 | -0.76 |
| SVRMHC | SQT | CMP | 8.77 | 7.11 | 8.97 | 6.52 |  | SVMHC | SQT | CMP | 1.72 | 0.57 | 1.79 | -0.70 |
| SVRMHC | SQT | CUF | 8.66 | 6.92 | 8.74 | 6.52 |  | SVMHC | SQT | CUF | 1.71 | 0.39 | 1.77 | -0.66 |
| SVRMHC | SQT | CSF | 8.76 | 7.14 | 8.85 | 6.51 |  | SVMHC | SQT | CSF | 1.74 | 0.29 | 1.77 | -0.68 |
| SVRMHC | SSU | CSP | 6.81 | 6.57 | 6.84 | 6.51 |  | SVMHC | SSU | CSP | -1.29 | -0.98 | -0.69 | -1.31 |
| SVRMHC | SSU | CDP | 6.70 | 6.56 | 6.78 | 6.51 |  | SVMHC | SSU | CDP | -1.46 | -1.10 | -0.80 | -1.48 |
| SVRMHC | SSU | CDB | 6.59 | 6.53 | 6.64 | 6.51 |  | SVMHC | SSU | CDB | -1.23 | -0.98 | -0.77 | -1.27 |
| SVRMHC | SSU | CMP | 6.70 | 6.54 | 6.75 | 6.51 |  | SVMHC | SSU | CMP | -1.42 | -1.00 | -0.77 | -1.52 |
| SVRMHC | SSU | CUF | 6.63 | 6.55 | 6.64 | 6.53 |  | SVMHC | SSU | CUF | -1.32 | -0.94 | -0.73 | -1.37 |
| SVRMHC | SSU | CSF | 6.66 | 6.55 | 6.69 | 6.53 |  | SVMHC | SSU | CSF | -1.38 | -0.94 | -0.76 | -1.42 |
|  |  |  |  |  |  |  |  |  |  |  |  |  |  |  |
| XSCORE | SRW | CSP | 7.92 | 7.97 | 8.36 | 7.78 |  |  |  |  |  |  |  |  |
| XSCORE | SRW | CDP | 7.79 | 7.77 | 8.29 | 7.45 |  |  |  |  |  |  |  |  |
| XSCORE | SRW | CDB | 7.97 | 7.81 | 8.09 | 7.63 |  |  |  |  |  |  |  |  |
| XSCORE | SRW | CMP | 7.84 | 7.66 | 8.23 | 7.38 |  |  |  |  |  |  |  |  |
| XSCORE | SRW | CUF | 7.66 | 7.63 | 7.99 | 7.22 |  |  |  |  |  |  |  |  |
| XSCORE | SRW | CSF | 7.97 | 7.87 | 8.27 | 7.69 |  |  |  |  |  |  |  |  |
| XSCORE | SLR | CSP | 12.29 | 9.67 | 12.55 | 7.83 |  |  |  |  |  |  |  |  |
| XSCORE | SLR | CDP | 12.11 | 9.51 | 12.48 | 7.81 |  |  |  |  |  |  |  |  |
| XSCORE | SLR | CDB | 11.97 | 9.30 | 12.09 | 7.96 |  |  |  |  |  |  |  |  |
| XSCORE | SLR | CMP | 12.30 | 9.23 | 12.52 | 7.77 |  |  |  |  |  |  |  |  |
| XSCORE | SLR | CUF | 12.63 | 9.23 | 12.69 | 7.91 |  |  |  |  |  |  |  |  |
| XSCORE | SLR | CSF | 12.14 | 9.16 | 12.46 | 7.74 |  |  |  |  |  |  |  |  |
| XSCORE | SBT | CSP | 12.23 | 9.55 | 12.29 | 7.84 |  |  |  |  |  |  |  |  |
| XSCORE | SBT | CDP | 12.27 | 9.25 | 12.50 | 7.75 |  |  |  |  |  |  |  |  |
| XSCORE | SBT | CDB | 11.68 | 9.43 | 12.03 | 7.96 |  |  |  |  |  |  |  |  |
| XSCORE | SBT | CMP | 12.41 | 9.69 | 12.66 | 7.77 |  |  |  |  |  |  |  |  |
| XSCORE | SBT | CUF | 12.47 | 9.32 | 12.71 | 7.89 |  |  |  |  |  |  |  |  |
| XSCORE | SBT | CSF | 12.39 | 9.53 | 12.59 | 7.89 |  |  |  |  |  |  |  |  |
| XSCORE | SRD | CSP | 7.80 | 7.53 | 8.13 | 7.38 |  |  |  |  |  |  |  |  |
| XSCORE | SRD | CDP | 7.96 | 7.72 | 8.16 | 7.48 |  |  |  |  |  |  |  |  |
| XSCORE | SRD | CDB | 7.44 | 7.64 | 7.70 | 7.26 |  |  |  |  |  |  |  |  |
| XSCORE | SRD | CMP | 7.24 | 7.77 | 8.13 | 7.13 |  |  |  |  |  |  |  |  |
| XSCORE | SRD | CUF | 7.75 | 7.72 | 8.18 | 7.54 |  |  |  |  |  |  |  |  |
| XSCORE | SRD | CSF | 7.43 | 7.83 | 8.22 | 7.36 |  |  |  |  |  |  |  |  |
| XSCORE | SBP | CSP | 9.34 | 10.82 | 12.04 | 7.86 |  |  |  |  |  |  |  |  |
| XSCORE | SBP | CDP | 12.25 | 10.88 | 12.58 | 7.68 |  |  |  |  |  |  |  |  |
| XSCORE | SBP | CDB | 10.43 | 10.29 | 11.62 | 7.87 |  |  |  |  |  |  |  |  |
| XSCORE | SBP | CMP | 11.25 | 11.29 | 12.31 | 7.63 |  |  |  |  |  |  |  |  |
| XSCORE | SBP | CUF | 10.25 | 11.21 | 12.44 | 7.71 |  |  |  |  |  |  |  |  |
| XSCORE | SBP | CSF | 11.69 | 11.05 | 12.27 | 7.78 |  |  |  |  |  |  |  |  |
| XSCORE | SQT | CSP | 12.65 | 9.72 | 12.72 | 7.85 |  |  |  |  |  |  |  |  |
| XSCORE | SQT | CDP | 12.38 | 9.79 | 12.50 | 7.80 |  |  |  |  |  |  |  |  |
| XSCORE | SQT | CDB | 12.29 | 10.02 | 12.29 | 7.75 |  |  |  |  |  |  |  |  |
| XSCORE | SQT | CMP | 12.63 | 9.95 | 12.75 | 7.86 |  |  |  |  |  |  |  |  |
| XSCORE | SQT | CUF | 12.72 | 9.80 | 12.76 | 7.70 |  |  |  |  |  |  |  |  |
| XSCORE | SQT | CSF | 12.72 | 10.44 | 12.79 | 7.79 |  |  |  |  |  |  |  |  |
| XSCORE | SSU | CSP | 7.45 | 7.74 | 7.83 | 7.29 |  |  |  |  |  |  |  |  |
| XSCORE | SSU | CDP | 7.55 | 7.85 | 7.97 | 7.36 |  |  |  |  |  |  |  |  |
| XSCORE | SSU | CDB | 7.76 | 7.55 | 7.94 | 7.17 |  |  |  |  |  |  |  |  |
| XSCORE | SSU | CMP | 7.54 | 7.63 | 8.07 | 7.27 |  |  |  |  |  |  |  |  |
| XSCORE | SSU | CUF | 7.89 | 8.07 | 8.27 | 7.63 |  |  |  |  |  |  |  |  |
| XSCORE | SSU | CSF | 7.62 | 7.62 | 7.83 | 7.39 |  |  |  |  |  |  |  |  |

The median over the last 10 generations indicates a rough estimation for the convergence of the population while the median over the first 10 generations gives a rough estimation of the speed of optimization in comparison to other operator combinations. Only the data for the “single point amino acid mutation” is shown since the results for the “single point nucleobase mutation” were similar. SRW: roulette wheel selection, SLR: linear rank selection, SBT: binary tournament selection, SRD: random selection, SBP: best percent selection, SQT: q tournament selection, SSU: stochastic universal sampling selection. CSP: single point cross over, CDP: double point cross over, CDB: distance biosector cross over, CMP: multiple point cross over, CUF: uniform cross over, CSF: shuffle cross over.
